# Removal of Six Esophageal Metals in a Four-Month-Old Infant: A Rare Case

**DOI:** 10.7759/cureus.52024

**Published:** 2024-01-10

**Authors:** Vikas Jain, Riya Jain, Rishabh Jain, Anshuman Chauhan, Nitin Kotwal

**Affiliations:** 1 Surgery, B. J. Hospital & Research Institute, Gondia, IND; 2 College of Medicine, Jawaharlal Nehru Medical College, Wardha, IND; 3 College of Medicine, Seth GS Medical College, Mumbai, IND; 4 Pediatric Critical Care Medicine, B. J. Hospital & Research Institute, Gondia, IND; 5 Anaesthesiology, B. J. Hospital & Research Institute, Gondia, IND

**Keywords:** infant, choking, diagnosis, endoscopy, esophagus, foreign body

## Abstract

This case report details the distinctive and demanding clinical situation involving a four-month-old female neonate. Her chief complaint was a two-day refusal to consume food orally, accompanied by episodes of vomiting following feedings and a sensation of choking in the throat. The referring physician suspected the presence of a foreign body in the patient's esophagus and advised a chest X-ray following a thorough examination. The presence of six hyperdense metallic foreign bodies in the upper, mid, and distal dorsal esophagus was confirmed by subsequent CT thorax imaging. This case was managed through the implementation of a multidisciplinary strategy. A decision was reached to conduct an endoscopic assessment; a substantial foreign object resembling a boulder was detected throughout the procedure, resulting in total obstruction of the esophageal lumen. Despite some challenges, this foreign object was effectively extracted by utilizing a Dormia basket. After that, endoscopy was used to detect five more metallic foreign bodies, all successfully eliminated endoscopically. The postoperative course was characterized by the 24-hour prophylactic Ryles tube insertion, followed by the resumption of breastfeeding. The infant's recovery and positive attitude on the second day following the operation indicate the case's successful resolution, emphasizing the criticality of a timely intervention in similar circumstances. This report underscores the clinical management and treatment of multiple metallic foreign bodies in a pediatric patient while also stressing the importance of prompt diagnosis and interprofessional collaboration in complex and exceptional cases.

## Introduction

Ingestion of a foreign object is a rare but challenging medical circumstance, particularly for babies and young children still developing their immune systems. A female baby who was four months old at the time was brought to our medical facility by her perceptive referring physician with a clinical presentation, which was both unusual and outstanding. This case demonstrates the unique characteristics of foreign body ingestion in infants and toddlers and the critical significance of a prompt diagnosis and therapy that follows.

Foreign bodies are swallowed by infants and toddlers much less frequently than by older children and adults. However, when it does occur, the inability of these young infants to communicate adequately and the possibly lethal repercussions of having a foreign body lodged in their digestive tract constitute a substantial clinical challenge. This instance demonstrates how important clinical competence and a collaborative effort between other disciplines can be when addressing the challenges posed by such situations.

A referral from a doctor was the first action taken toward arriving at a diagnosis and determining a course of treatment. Due to the doctor's intense concern that a foreign object was lodged somewhere in the patient's upper gastrointestinal tract, he recommended that the patient get a chest X-ray as soon as possible after recognizing the severity of the patient's symptoms. The results of additional diagnostic tests, including a CT thorax, revealed the presence of many metallic foreign things within the esophagus, which necessitated immediate action on the part of the medical staff. Cooperation between the doctor who referred this patient to us and our medical personnel was essential in efficiently managing this case.

In the following sections, we will talk about the complete clinical presentation, the diagnostic workup, the challenges faced when endoscopically removing these foreign materials, and the postoperative recovery of the newborn. This report illustrates the value of teamwork between primary care physicians and specialists, fast action, and the excellent outcome. In particular, the case presented a unique clinical complexity.

## Case presentation

The primary subject of this case report is a female infant currently four months old. Because she had been vomiting for two days straight, her parents decided to bring her to our medical center for treatment. The child's parents reported that their infant would frequently choke after eating and rapidly throw up afterwards. The intensity of the symptoms prompted prompt efforts to locate and consult a physician.

Brief medical history and clinical presentation

In female infant medical history, there were no instances that were deemed to be serious medical disorders or models of foreign body ingestion, and before this event, the infant's first few years had passed without any major health concerns being reported.

The rapid onset of symptoms characterized the clinical presentation, mostly connected to eating. Her mother and father were concerned about the female infant's resistance to taking anything by mouth, even food. The infant appeared to be in great distress whenever anyone attempted to feed her, and she frequently threw up severely immediately after consuming food. Because of the remarkable force with which the newborn was regurgitating its food, there was a cause for concern over the infant's health and nutrition.

The female infant parents saw, as well, that their daughter frequently experienced choking episodes following meals. They were already in much pain, but this terrifying sign made it even worse, so they headed to the hospital immediately.

In addition to these troubling symptoms, the female infant did not exhibit any signs of disease, such as a temperature or difficulty breathing. It was determined that she was a healthy infant when she was brought in for evaluation, and she did not exhibit any apparent signs of respiratory distress or pain other than those she had previously experienced after oral intake.

Even though no clear external reasons or predisposing medical illnesses were present, the clinical findings and reported symptoms prompted a thorough assessment to discover the underlying source of the infant's pain. This was done even though there were no evident external causes. The female infant required more diagnostic testing and the combined efforts of a team to discover the source of her symptoms so that they could be treated in an efficient and timely manner.

Diagnostic assessment

CT Thorax Findings

**Figure 1:**
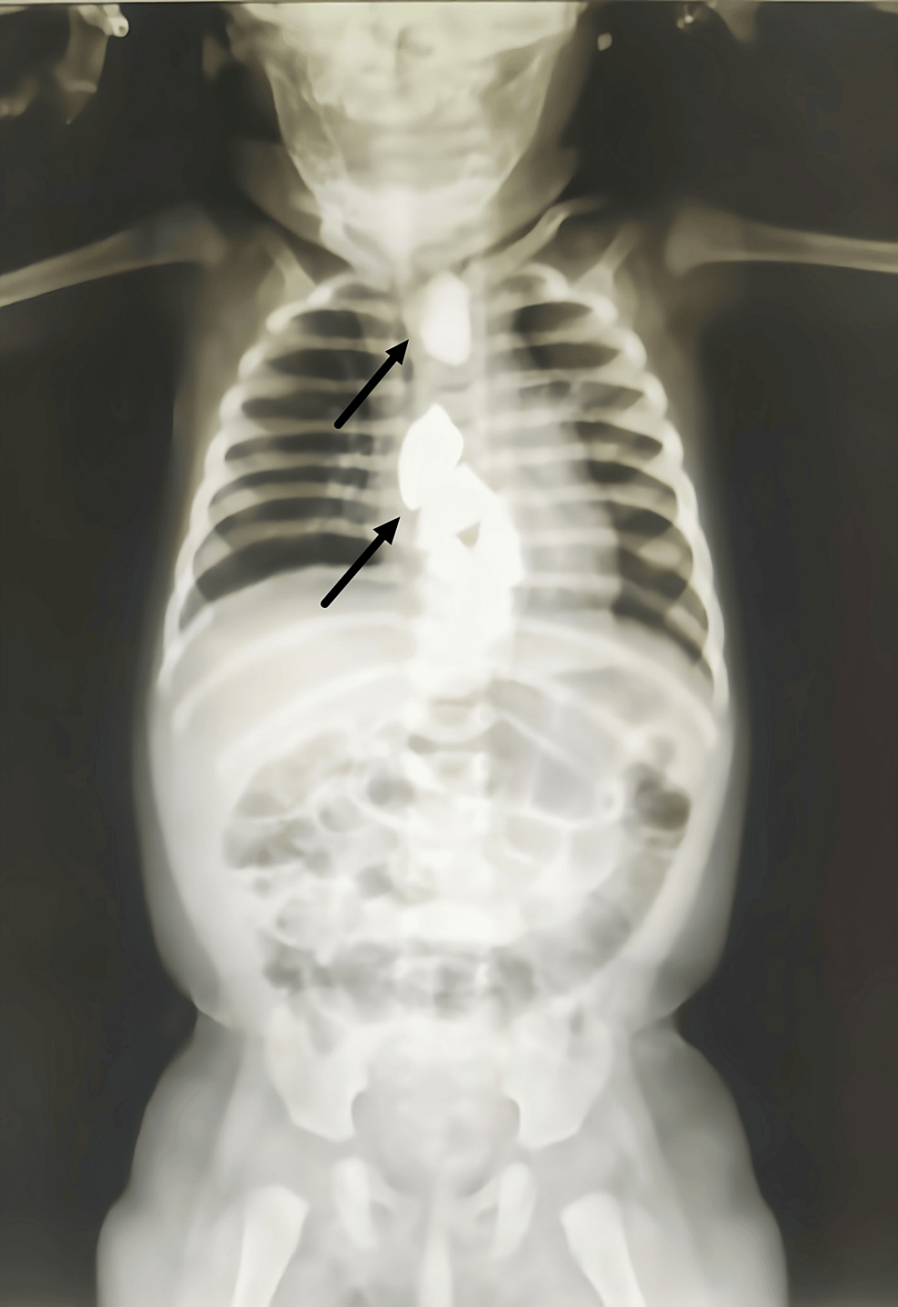
Chest X-ray image

First, an X-ray of the female infant's chest was taken as the referring physician wisely suggested to understand her condition better. The X-ray images in Figure 1 may point to the foreign items in the patient's esophagus, stomach, or both. In light of the findings from these X-rays, a CT thorax examination was carried out to offer a more in-depth analysis of the potential foreign bodies.

**Figure 2:**
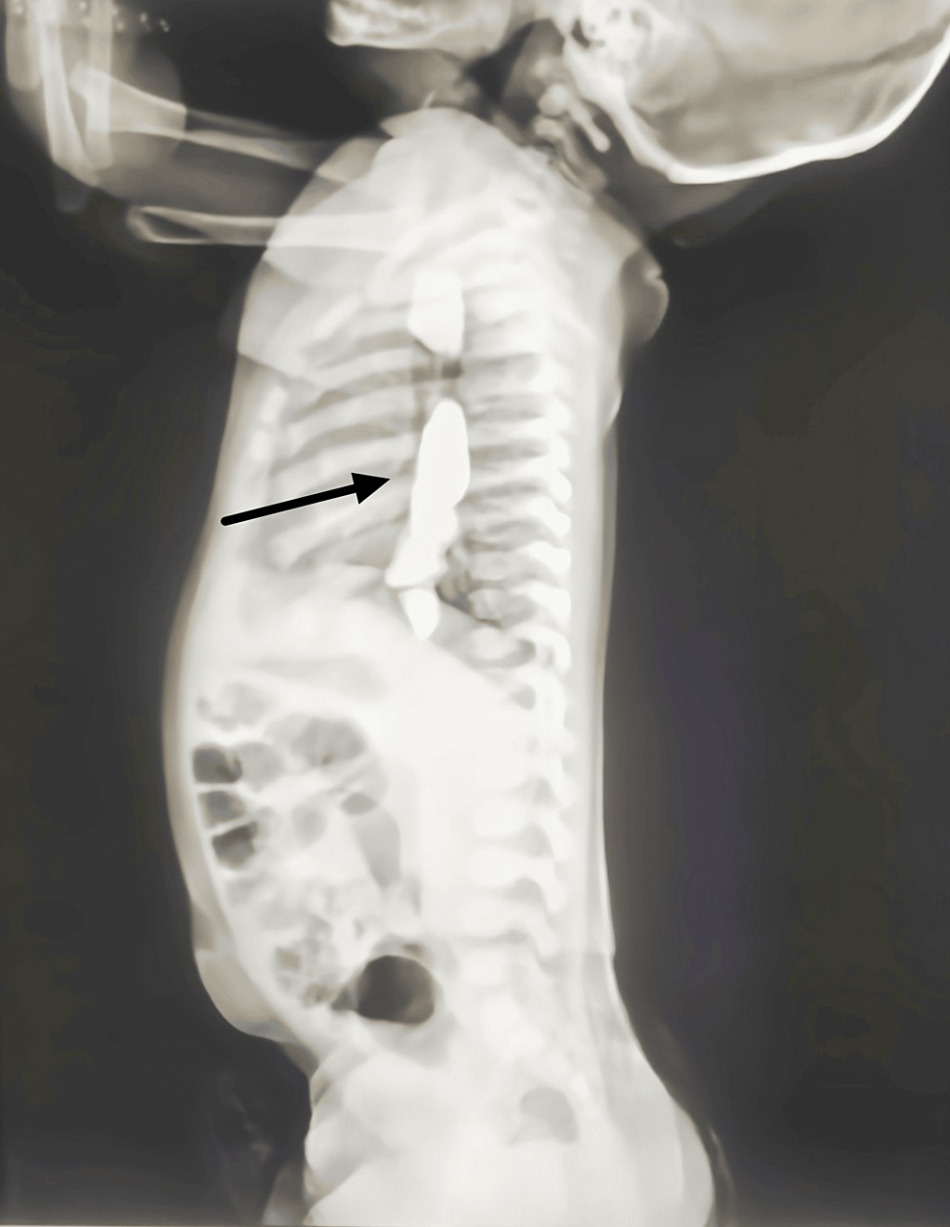
CT thorax image of the esophageal foreign bodies

**Figure 3:**
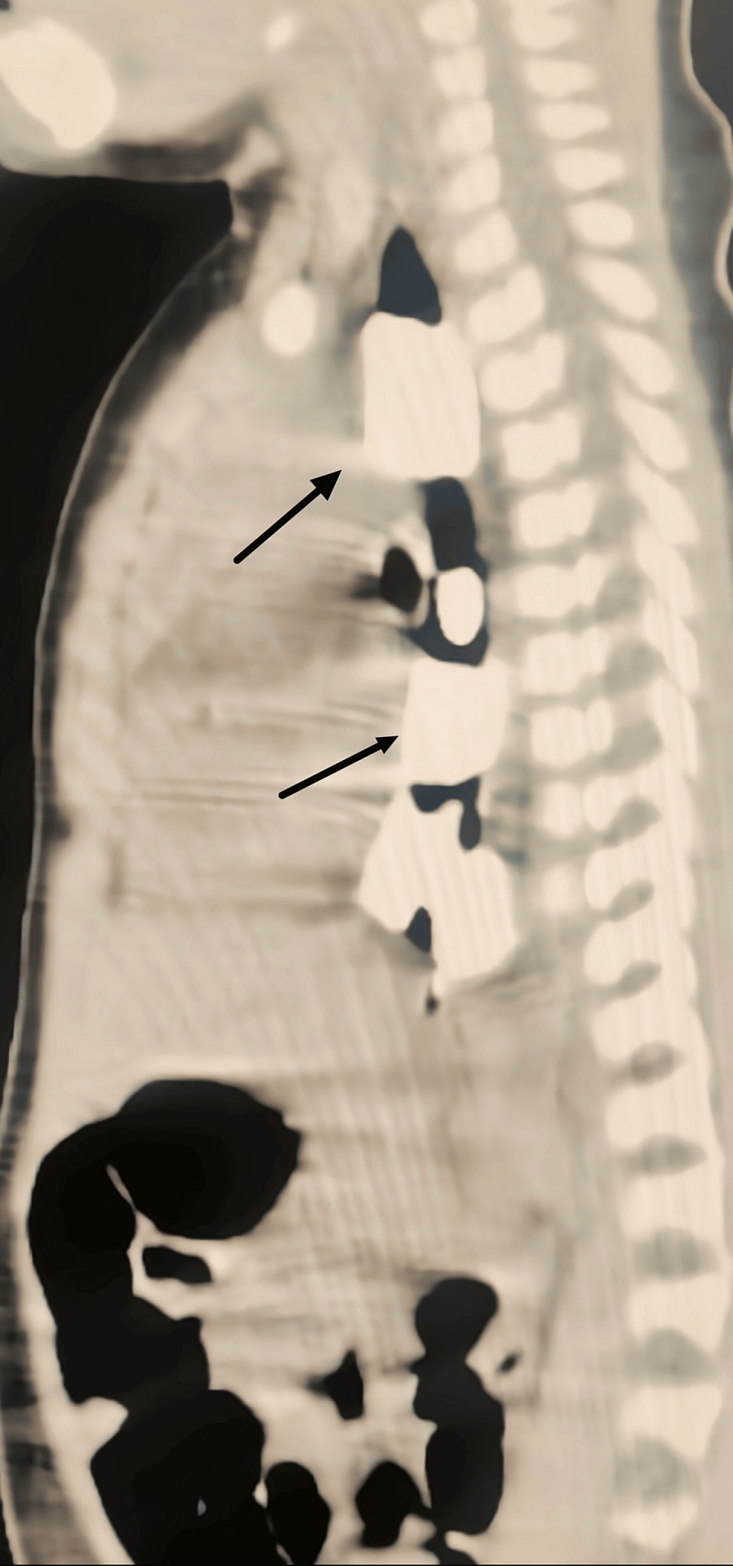
CT thorax image of the metallic foreign bodies

An in-depth look at the esophagus, from its proximal to its distal dorsal parts, was caught by the thorax CT scan, as seen in Figure 2 and Figure 3. The pictures revealed the presence of six distinct hyperdense structures, the largest of which was around 2 millimeters in length and 1.3 millimeters in width. These strata were found to contain a metallic density of up to 2,000 Hounsfield units (HU), which was detected. To decide whether or not to execute an intervention to avert an impaction, it was essential to be aware of where the foreign bodies were positioned within the esophagus. 

Radiopacity, as seen on the CT scan, differentiated the foreign bodies from the normal esophageal tissues and structures. With these results, the next course of therapy was decided upon, which likely involved an endoscopic examination and the removal of a foreign body.

Throughout the diagnostic process, the chest X-ray and CT thorax were essential in verifying foreign bodies' presence in the upper gastrointestinal tract. In addition, these imaging techniques provided important information on the location of the foreign bodies and their size and density. These particulars were essential in forming the treatment approach and paving the way for this unusual clinical scenario's prompt and efficient management.

Management and treatment

Decision for Endoscopic Evaluation

As soon as the CT thorax indicated the presence of many metallic foreign bodies in the patient's upper, middle, and distal dorsal esophagus, the decision to perform an endoscopic assessment was made as quickly as possible. Because a foreign body being lodged in the esophagus, especially in a youngster, might have serious repercussions, quick medical intervention was required in this case. After some consideration, it was decided that an endoscopic examination was needed to identify the foreign bodies and devise a strategy for removing them.

Surgical Procedure and Difficulties Faced

The subsequent stage consisted of bringing a female infant into the operating room so that an endoscopic surgery could be performed. Endoscopy was performed on the patient under general anesthesia, which enabled the foreign bodies to be seen more clearly throughout the procedure. The large foreign mass, which resembled a rock and was found to be lodged in the post-cricoid region, was the primary focus of the surgery because it was entirely blocking the lumen of the esophagus. The foreign body was located using a dormia basket, which needed delicate manipulation by the medical professional. This procedure required treatment that was both delicate and deft because of the size and location of the foreign body.

**Figure 4:**
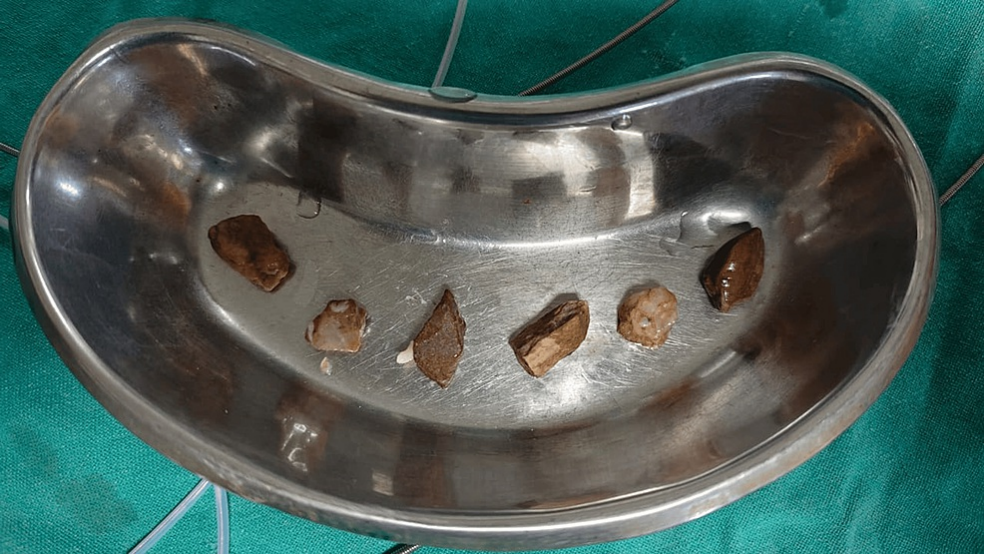
Removed smaller foreign bodies

**Figure 5:**
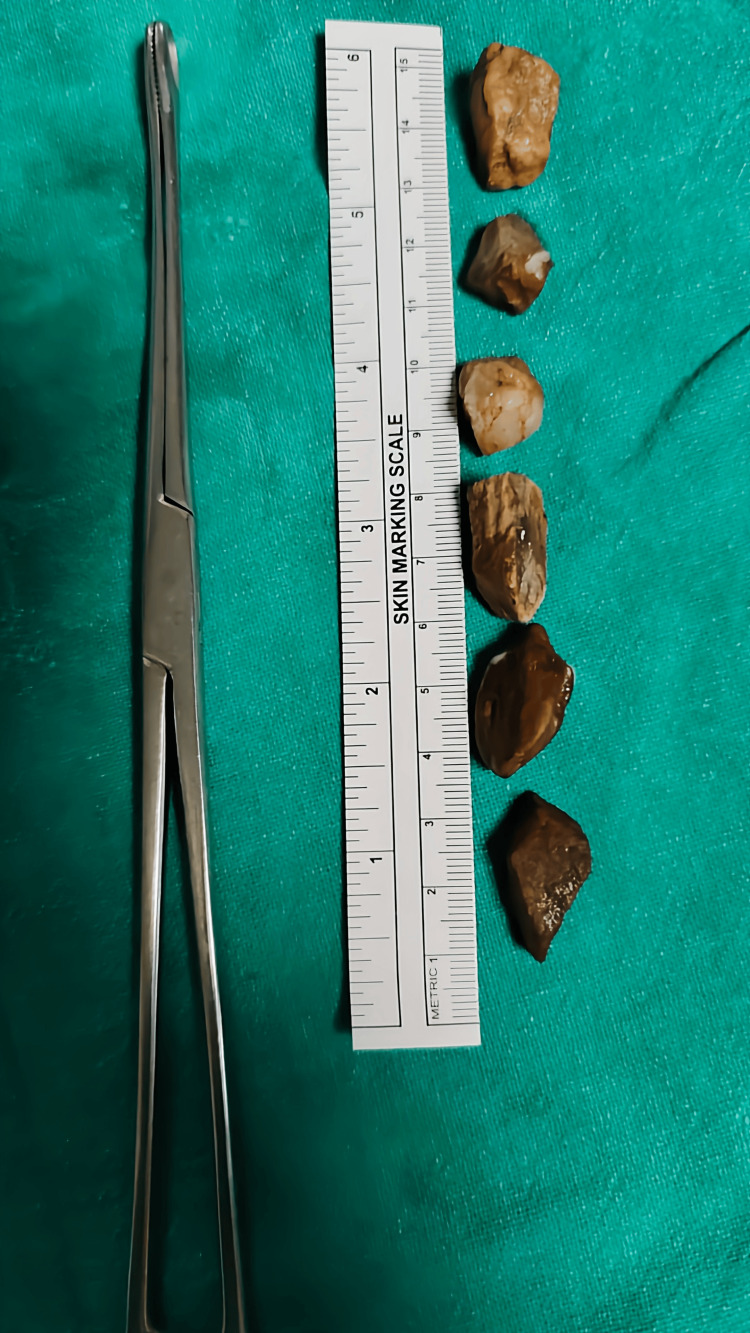
Foreign body removal

The discovery of five more metallic foreign things (shown in Figure 4 and Figure 5) in the esophagus made the treatment more challenging than it would have been otherwise. Endoscopic procedures were used to remove the remaining foreign entities one at a time after the initial big foreign body was withdrawn. If an endoscopic evaluation had not been undertaken to find and remove foreign bodies from the newborn patient's esophagus, the risks and effects of esophageal foreign body impaction would have been significantly more severe.

Placement of the NG Tube

After the operation, a nasogastric (NG) tube was inserted for preventive purposes and left in place for 24 hours. The esophageal mucosa was allowed to heal once this precautionary measure was taken, ensuring that adequate feeding was resumed and the mucosa could recover. After this, the infant was able to begin nursing, which facilitated a significant acceleration in the healing process that had been previously underway.

Follow-up and outcome

Patient's Progress and Recovery Following the Surgery

The female infant's clinical status significantly improved in the immediate postoperative period after numerous metallic foreign bodies were extracted endoscopically from her esophagus. Within the first day after the operation, the female infant's demeanor saw a significant shift for the better. Her distressing symptoms, which included reluctance to eat, feelings of choking, and episodes of vomiting, gradually improved over time.

The baby exhibited evidence of returning to her usual, cheerful, and active self as early as the second day following surgery, only two days after the procedure. Because the foreign bodies were removed from the newborn's esophagus, the uncomfortable symptoms that the infant was experiencing were alleviated as a result of the intervention, which was successful and had a positive impact on the well-being of the infant.

Timing of Discharge

The patient was discharged from the hospital on the third day after the operation after a choice to do so was made. It turned out that the newborn had a speedy recovery and could return to being breastfed; thus, it was decided that the treatment should be scheduled for now. The favorable postoperative course that the infant displayed, highlighted by a speedy improvement in her condition, contributed to the decision to discharge the infant from the hospital.

Mention of Follow-up Appointments or Interventions

A structured follow-up program was developed as a part of the postoperative treatment that the female infant would get. This tactic aimed to maintain a close track of the infant and monitor how well she was recovering from her injuries. To provide each patient with the best possible medical care, the scheduling and frequency of their appointments have been determined.

Suppose a newborn has consumed a foreign body, in that case, planning regular and in-depth follow-up appointments is imperative to examine the infant's overall health and the esophagus' ability to function and detect and treat any potential late-onset disorders. Checkups consistently allow the medical personnel to track a patient's condition over time and evaluate their development.

## Discussion

This female infant's case presents a novel and challenging clinical scenario because she is a newborn baby who is only four months old and has swallowed several metallic foreign bodies. Due to the extreme rarity of many foreign bodies in such a young child, a dialogue must be had regarding the consequences, possible causes, and significance of prompt detection and care.

Uniqueness of the case

The most significant aspect of this case is the discovery of several metallic foreign entities in the esophagus of a newborn child who was just four months old. It is exceedingly unusual for an infant as young as four months to have many foreign bodies in the upper gastrointestinal system. Ingestion of foreign bodies is common among children, especially toddlers. However, it is exceptionally unusual for an infant to have multiple foreign bodies in the upper gastrointestinal system. The atypical presentation of this case, along with its challenges in terms of diagnosis and treatment, distinguishes it as a unique and noteworthy instance.

Multiple foreign bodies in a newborn of this age are statistically fascinating. Still, they also illustrate the importance of remaining cautious and skeptical even in patients not typical of the investigated condition. This example exemplifies the significance of maintaining a high index of suspicion for the possibility of a foreign body being ingested by infants of any age and highlights the importance of timely detection and treatment.

Related case studies

New methods for removing foreign bodies from a child's digestive tract are the focus of Choe et al.'s investigation [1]. The feasibility of using a Foley catheter or magnet tube to expel foreign bodies from the digestive tract was explored. This research should lead to novel, less intrusive methods for treating foreign body ingestion in children. The authors hope to add to the corpus of knowledge on foreign body removal in children by investigating these procedures, which may provide safer and more effective approaches to dealing with such occurrences.

Within the scope of this work, we present a case study that details the successful extrication of a foreign body. Extricating an impacted metallic cap from the esophagus, which had become a sharp foreign body, was the objective of this particular procedure. The removal of foreign bodies is a technically complex process, yet the circumstances of this incident highlight the significance of prompt and efficient intervention when dealing with potentially hazardous objects. Sarem et al.'s research [2] offers insightful knowledge regarding the approaches that are taken to treat this kind of foreign body ingestion and the challenges that may be encountered and the outcomes that may be anticipated.

Yuan et al.'s research [3] investigated the practice of removing foreign bodies from the esophagus, stomach, and small intestine using endoscopy. The findings are reported from a comprehensive sample size of 846 cases conducted in China, making this a trustworthy resource. The primary emphasis is placed on endoscopic procedures for the removal of foreign bodies. The purpose of this research is to gain an understanding of the rates of success, complications, and outcomes associated with endoscopic procedures performed in clinical settings. It is an excellent resource for learning the ins and outs of endoscopic foreign body removal, including all the nuts and bolts.

Potential causes and risk factors

How could a newborn only four months old get many metals foreign bodies lodged in their stomach? Several ideas could be investigated even though the underlying reason is still unknown. Infants of this age are at a developmental stage where they explore their surroundings by putting things in their mouths. During this stage, they are also in the process of teething. When this finding was being made, infants might have accidentally consumed these pieces of metal. Exposure to the environment is yet another potential determining factor. Infants are frequently seen in close contact with objects, toys, and other items the infants could consume. It is possible that the infant grasped the foreign entities and swallowed them without realizing what they were. The fact that the parents cannot recall the specific circumstances surrounding the ingestion underscores the importance of maintaining vigilance to prevent access to small objects, particularly ones that may not appear to be dangerous at first glance.

Importance of timely diagnosis and intervention

Ingestion of foreign bodies, like the one that infant experienced in this case, brings to light the critical importance of timely identification and care. Ingestion of a foreign body in newborns and young children can lead to severe complications, such as airway obstruction, injury to the esophagus, and even mediastinitis. A delay in identification or treatment, which may otherwise prevent potentially catastrophic outcomes, can make these dangers even more severe.

In this particular instance, timely intervention was primarily secured because the referring doctor detected the clinical symptoms early on and prudently decided to begin the diagnostic assessment. This allowed for a rapid treatment to be administered. The radiographic data from an X-ray of the chest and then a CT scan of the thorax revealed a specific localization and characterization of the alien entities, which allowed for educated decision-making regarding the therapeutic plan.

The fast restart of nursing and the effective removal of a number of metallic foreign bodies by endoscopy are examples of the prompt action that contributed to this outstanding outcome. The benefits of a holistic point of view, interdisciplinary teamwork, and a patient-centered approach are demonstrated here.

The female infant's tale is unique and illuminating because it demonstrates the impact that something unfamiliar may have on a young child. Because of the patient's distinctive presentation, they need close monitoring of their health and fast treatment to avoid complications. The case study highlights the critical role that healthcare practitioners have in the positive outcome of their patients' ailments, even in unusual and hard situations.

Her parents belong to a sub-urban area and not well financially sound, but they were very happy to see her daughter recovering well.

## Conclusions

A four-month-old infant presented a novel and demanding therapeutic scenario when it was revealed that she had numerous metallic foreign particles lodged in her esophagus, but she responded well to therapy. The positive outcome of the patient was a consequence of a multidisciplinary team's efforts, which included a prompt diagnosis and prompt treatment. Rapid analysis and intervention characterized this initiative.The discovery that such a young child has multiple metallic foreign bodies is unusual in this situation. The rapid improvement in the infant's condition after the foreign bodies were extracted from their digestive tract via endoscopy proves that the medical intervention was successful.

It was essential in this situation for those in the medical field to collaborate. From the acute clinical judgement of the referring physician, Dr. Anshuman Chouhan, through the surgical skills of the endoscopist, this case highlights the need for a coordinated and multidisciplinary approach in challenging medical situations. The quick recovery of the newborn can be credited, in a large part, to the exceptional communication and coordination among the medical staff members who worked on their behalf. This case indicates that medical professionals should always be on the watch for foreign body ingestion, particularly in patient demographics that are not typical of those they treat. In addition, the significance of diagnostic imaging procedures, such as chest X-rays and CT scans of the thorax, in determining the most appropriate treatment strategy is underlined.

The positive outcome achieved for this female infant is a testament to the benefits of early diagnosis, a collaborative approach, and the expertise and experience of healthcare experts, particularly in unusual and complex cases. This challenging situation was ultimately resolved because of the patient's dogged determination and the unwavering commitment of the medical team.
